# Key transition technology of ski jumping based on inertial motion unit, kinematics and dynamics

**DOI:** 10.1186/s12938-023-01087-x

**Published:** 2023-03-02

**Authors:** Jinglun Yu, Xinying Ma, Shuo Qi, Zhiqiang Liang, Zhen Wei, Qi Li, Weiguang Ni, Shutao Wei, Shengnian Zhang

**Affiliations:** 1 Key Laboratory of Exercise and Health Sciences of Ministry of Education, Shanghai University of Sport, Shanghai, 200438 China; 2 School of Exercise and Health, Shanghai University of Sport, Shanghai, China; 3 Foundation Courses Research Center, Silicon Lake College, Kunshan, China; 4 Physical Education College, Jilin University, Changchun, China; 5 361 Degree Co., Ltd., Xiamen, China

**Keywords:** Ski jumping, Technology, Kinematics, Dynamics, IMU, Measurement systems

## Abstract

**Background:**

The development and innovation of biomechanical measurement methods provide a solution to the problems in ski jumping research. At present, research on ski jumping mostly focuses on the local technical characteristics of different phases, but studies on the technology transition process are less.

**Objectives:**

This study aims to evaluate a measurement system (i.e. the merging of 2D video recording, inertial measurement unit and wireless pressure insole) that can capture a wide range of sport performance and focus on the key transition technical characteristics.

**Methods:**

The application validity of the Xsens motion capture system in ski jumping was verified under field conditions by comparing the lower limb joint angles of eight professional ski jumpers during the takeoff phase collected by different motion capture systems (Xsens and Simi high-speed camera). Subsequently, the key transition technical characteristics of eight ski jumpers were captured on the basis of the aforementioned measurement system.

**Results:**

Validation results indicated that the joint angle point-by-point curve during the takeoff phase was highly correlated and had excellent agreement (0.966 ≤ *r* ≤ 0.998, *P* < 0.001). Joint root-mean-square error (RMSE) differences between model calculations were 5.967° for hip, 6.856° for knee and 4.009° for ankle.

**Conclusions:**

Compared with 2D video recording, the Xsens system shows excellent agreement to ski jumping. Furthermore, the established measurement system can effectively capture the key transition technical characteristics of athletes, particularly in the dynamic changes of straight turn into arc in inrun, the adjustment of body posture and ski movement during early flight and landing preparation.

## Background

Ski jumping has a long history of development, and it has elicited increasing attention from researchers in recent years [1]. Ski jumping performance is frequently divided into four different phases: inrun, takeoff, flight (early and stable flight) and landing [2]. The wide range and long distance of motion scenes in sports make formulating experimental plans difficult. The severe outdoor environment during winter also challenges the collection of high-quality data. On the one hand, measurement accuracy should be considered for the measurement methods; on the other hand, the adverse effects of the measurement system on the athletes should be reduced [3]. During the primary stage of the study, these contradictory goals cannot be achieved simultaneously, and some trade-offs must be made in executing processes. However, with the development of science and technology, such as the innovation of instruments and equipment, the application of wireless transmission technology and the novel idea of applying inertial measurement units (IMUs) to ski jumping research [4], problems in research and measurement can gradually be solved. The characteristics of ski jumping necessitate that the technical movements of athletes are largely executed on the sagittal plane, making 2D video recording [5, 6] the primary research method. In recent years, IMUs have been widely used; they exhibit the advantages of high precision, small size and strong portability, making them the best choice for sports training and performance feedback [7–9]. Similarly, the use of wireless pressure insoles can more conveniently and economically collect the dynamic parameters of takeoff and landing without affecting athletes’ performance of technical movements [10, 11].

In the research on the biomechanics of ski jumping, most studies have focused on local technical characteristics at different phases, particularly takeoff and flight. Meanwhile, research on the transition process between different technologies (e.g. the process of straight turn into arc in inrun, the formation of ‘V’-shaped technology in early flight and preparations before landing) is less, and attention is low. However, ski jumping technology is continuously developing. Athletes can only achieve the best flight distance when they accurately complete the corresponding technical requirements and transition in each link. Arndt [12] pointed out that the technical mistakes made by ski jumpers in the previous phase (particularly in takeoff) cannot be remedied or eliminated through a flight phase technology. Therefore, building a whole or large measuring range for observing changes in athletes’ movement characteristics in continuous state and the transition process between different technologies seems necessary to deepen the understanding of athletes, coaches and researchers regarding ski jumping.

The objective of the current study was twofold. Firstly, it aimed to evaluate a measurement system (i.e. the merging of 2D video recording, IMU and wireless pressure insole) that can capture a wide range of sport performance in an outdoor experiment. Secondly, on the basis of the aforementioned research methods whilst observing the overall movement stage, this research focused on the key transition technical characteristics, including straight turn into arc in inrun, early flight in flight and landing preparation during landing. The measurement system was hypothesised to accurately obtain biomechanical parameters within a large range and the characteristic changes of key transition technologies.

## Methods

In an outdoor experiment, eight ski jumpers (from the Chinese national and provincial teams; male; age: 17.88 ± 0.83 years; height: 175.50 ± 3.07 cm; mass: 61.25 ± 2.12 kg; ski length: 236.88 ± 8.72 cm; ski brand: Slatnar; mean ± standard deviation) were equipped with an IMU-based motion capture suit with 17 sensors (240 Hz, Xsens MVN, the Netherlands) and wireless pressure insoles (50 Hz, Moticon, USA). Each IMU consisted of a 3D linear accelerometer, a 3D rate gyroscope and a 3D magnetometer. Each insole was composed of 13 pressure sensors with a measuring range of 0–50 N/cm^2^. Videos were recorded by seven cameras (200 Hz, 1200 × 800 dpi, Simi Motion, Deutschland) and two other cameras (500 Hz, 2048 × 2048 dpi, Fastcam Mini WX100, Japan). Climatic and environmental parameters were collected by a multifunctional weather station and a snow pack analyser (Snow Fork, Finland).

**Fig. 1:**
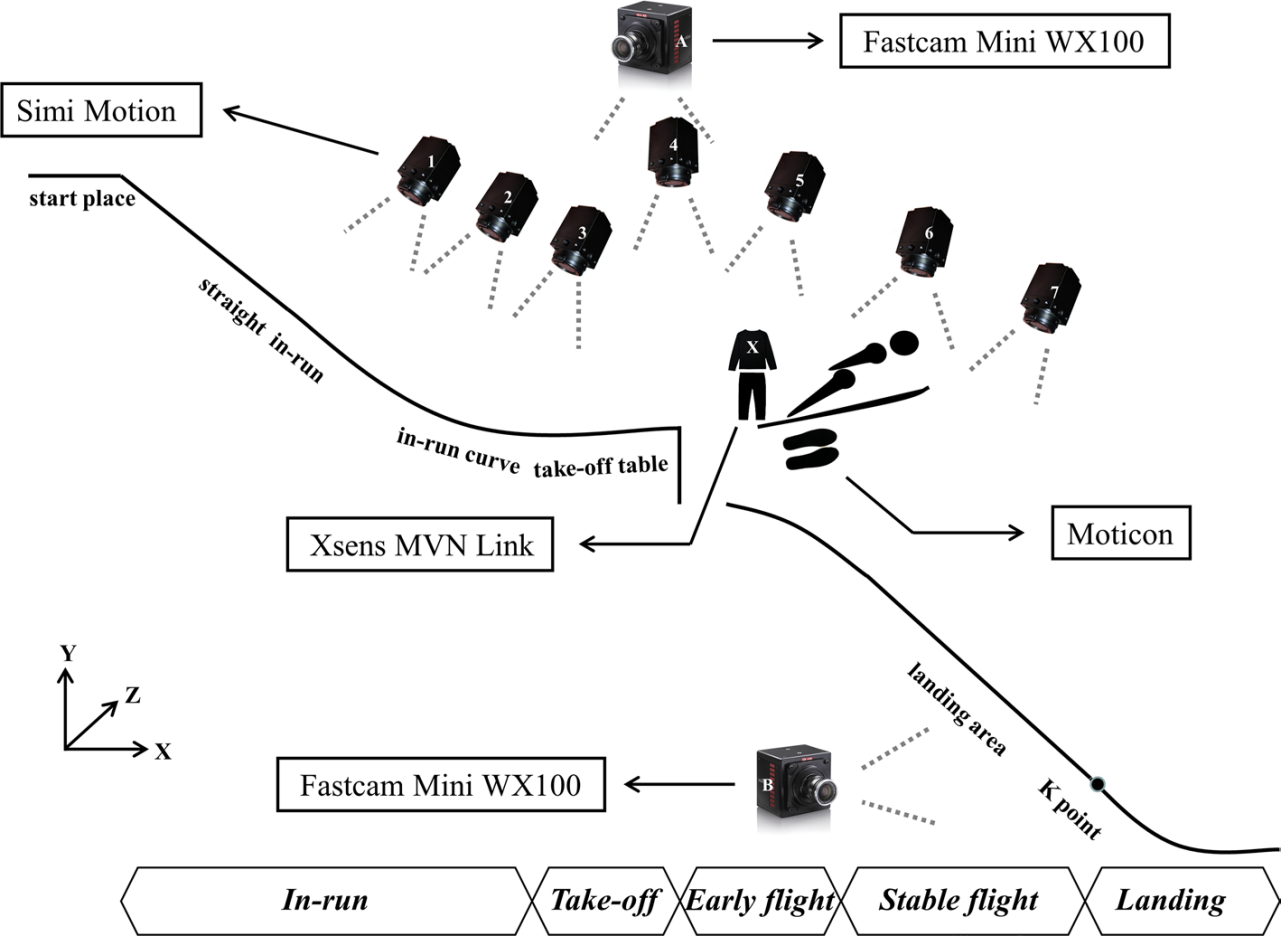
Equipment layout and movement phase division

The test conditions on the hill did not allow for 3D measurements, and thus 2D video recordings parallel to the sagittal plane were used. In the study design, seven Simi Motion high-speed cameras (Nos. 1–7) were selected and placed side by side on the table to record the technical movements of the ski jumpers in a long distance. Two Fastcam Mini WX100 high-speed cameras (A and B) were set up on one side of the takeoff table and landing area to focus on capturing the takeoff posture and landing buffer action of the ski jumpers. Meanwhile, the athletes wore Xsens suit and Moticon insole, and additionally, one IMU was placed on each of the two skis (Figs. 1 and 3a). The captured videos were stored on computer hard disk, and the Xsens and insole data were stored offline.

Definition of key transition technology. *Straight turn into arc in the inrun phase*: the inrun slideway consists of a straight area and an arc area, and evident transition points are found in the structure of the two areas (the straight turn into arc when entering this area and the arc turn into straight before entering the takeoff area). *Early flight in the flight phase*: early flight (the first stage of flight, within 0.50 s after takeoff [13]) is considered the key phase that affects flight distance, reflecting the quality of takeoff. *Landing preparation in landing phase*: landing and preparation before landing are critical to sports performance and safety. Bessone [14] proved that the landing preparation started around 0.36 s before the impact.

The test site was the normal hill of the Jilin Beidahu Snow Training Base, which meets the requirements of international snow competitions. The climatic and environmental parameters on the test day were as follows: wind speed was 1.3 ± 0.8 m/s; wind direction was westerly; temperature was − 2.4 ± 0.4 ℃; relative humidity (RH) was 27.6 ± 1.6%; CO2 was 437.3 ± 13.5 ppm; atmospheric pressure was 95.7 ± 0.0 kPa; for hardness, the track was 26.8 ± 1.2 N; natural snow was 7.8 ± 0.0 N and artificial snow was 15.3 ± 3.8 N. The shooting distance of the on-site measurement camera was 53 m (from the straight inrun area to the early flight area and landing area). Video shooting was calibrated with a 2D scale (length: 1 m), and each camera was calibrated with a black and white chessboard (size: 10 × 7; unit: 33.33 mm × 33.33 mm) for distortion. The Xsens and insole were measured during the entire process. Xsens completed posture calibration with npose + walk (standing still + walking), and the motion mode was single level.

The 2D coordinate system was defined as the *X*-axis, which was the sagittal axis (the horizontal line in the front and back directions), and the *Y*-axis, which was the vertical axis (the vertical line perpendicular to the horizontal line in the up and down directions). The 3D coordinate system was defined as the *Z*-axis, which was the forehead axis (the horizontal line in the left and right directions), and the *X*- and *Y*-axes were the same as those in the 2D condition. The joint definition was follows. Forward, up and outside were positive; backward, down and inside were negative. The video image and IMU data were synchronised with the characteristic time of the motion animation generated via Xsens software analysis. The IMU and insole data were synchronised with the captured acceleration value generated by the takeoff and landing actions.

The test procedure involved debugging the instruments and equipment on site in advance, including the camera’s erection position, shooting range, angle and aperture. Thereafter, all the instruments and equipment were marked before capturing, and climate and environmental parameters were collected in real time. The test firstly recorded the basic information of the athletes and the completed daily warm-up. Subsequently, the athletes were asked to wear the test equipment to adapt. All the athletes subjectively did not feel that the equipment exerted an effect on their movement. Finally, each athlete was allowed to complete two to three successful jumps within 1 h, and the coach would judge the quality. The interval between each jump was 15–20 min to reduce the influence of fatigue. The test climate and environmental conditions were stable. All the cameras had good visibility, and the conditions for each jump were the same as much as possible. All the experimental operators had biomechanical research background, and special staff members were responsible for assisting the athletes in wearing the equipment, capturing every jump and starting and closing Xsens and insoles.

All the captured videos were manually digitised by experienced technicians using Simi Motion software to obtain kinematic indicators, such as joint angle, centre of gravity velocity and position. Xsens kinematics data were analysed offline using Xsens-MVN Analyse 2020.0.2 software, imported offline as storage data through BodyPack and combined with calibration files to output kinematics indicators, such as joint angle and centre of mass position. Dynamics data were analysed using Moticon Science software to obtain dynamic indicators, such as plantar pressure distribution, acceleration and total pressure. Microsoft Office Excel 2021 was used for data sorting and summary. Origin 2017 64 bit and Microsoft Office PowerPoint 2021 were used for drawing. IBM SPSS Statistics 26 was used for statistical analysis and data processing.

In accordance with the characteristics of the ski jumping sport, the current research assumed that the body structure and technical characteristics of a ski jumper were symmetrical on the sagittal plane. The video data were based on the left side of the athletes, because the camera was located on the left side of the platform. Consequently, observing was easy and the motion image was not blocked. The athletes’ data at each stage were standardised (hundred differentiation), averaged and smoothed using a low-pass filter with a cutoff frequency of 8 Hz. To discuss the uniformity of video and IMU data under time series, curve correlation and root-mean-square error (RMSE) were selected for verification [15]. The following correlation thresholds were set for r, according to the guidelines given by Maria [16] and Hatamzadeh [17]: poor (< 0.5), moderate (≥ 0.5 and < 0.75), good (≥ 0.75 and < 0.9) and excellent (≥ 0.9). The results are displayed as descriptive statistics (mean ± standard deviation).

## Results

### Validation of measurement systems

**Fig. 2:**
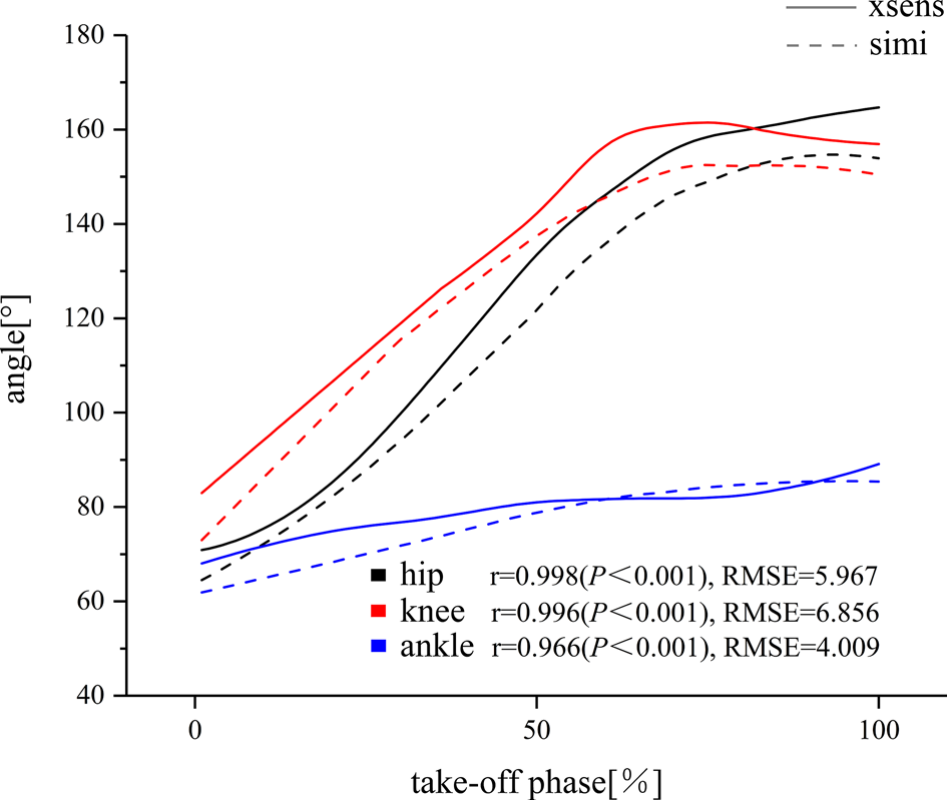
Example data of angle parameter from the same takeoff phase calculated using both methods. Angle (in the joint coordinate system of the left lower limbs, average of eight subjects) from the video data (Simi) and the merged IMU data (Xsens). The mode of joint motion is flexion and extension. Solid line = Xsens, lineation = Simi, black = hip, red = knee, blue = ankle

In accordance with the statistical analysis, the correlation coefficients and p-values for the joint angle point-by-point curve correlations (eight participants) measured by the two measurement systems (Simi-merged Xsens) during the takeoff phase were highly correlated and significant (*r*_*hip*_ = 0.998; *r*_*knee*_ = 0.996; *r*_*ankle*_ = 0.966, *P* < 0.001). Validation results show that the proposed system yields excellent statistical agreement (0.966 ≤ *r* ≤ 0.998). Joint RMSE differences between model calculations based on the two measurement systems were 5.967° for hip, 6.856° for knee and 4.009° for ankle (Fig. 2).

### Key transition technology

**Fig. 3:**
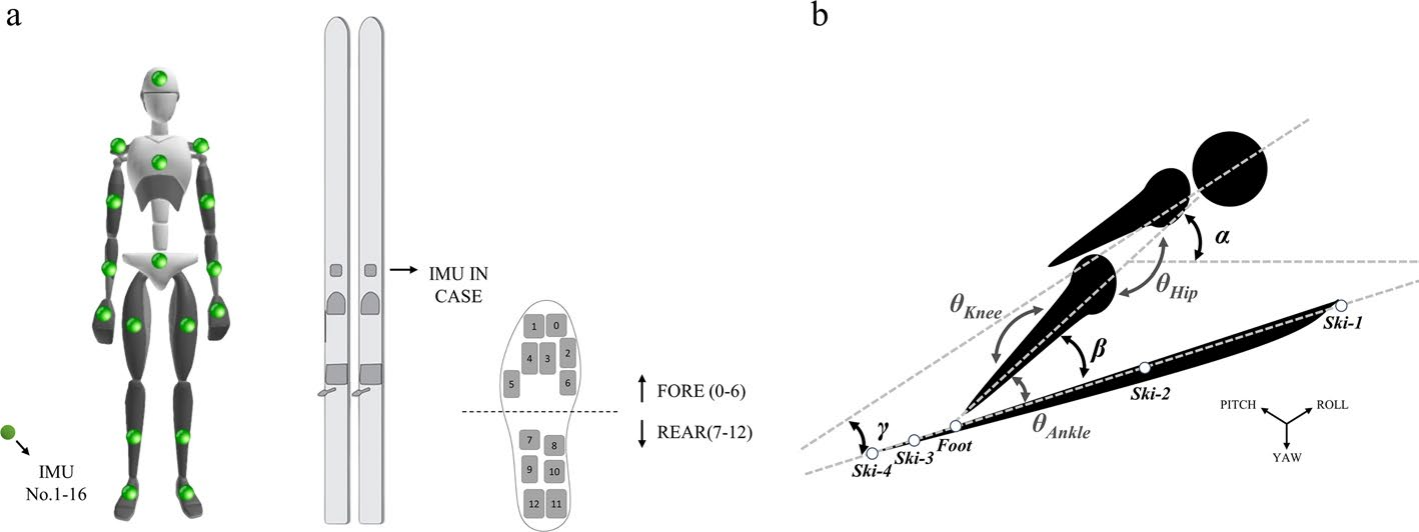
Graph **a** shows the placement position of the IMUs (Xsens, Nos. 1–16), total of 18 IMUs (with 2 others on the ski). Detecting area division of the fore (0–6) and rear (7–12) foot parts in a wireless pressure insole. Graph **b** presents the key angle parameters of an athlete’s body posture during early flight. α, the angle of attack, is the angle between the longitudinal axis of the body and the horizontal plane of the centre of gravity. β is the angle between the longitudinal axis of the body and the ski. γ is the angle between the upper part of the body. Points ski-1–4 constitute the whole ski

**Fig. 4:**
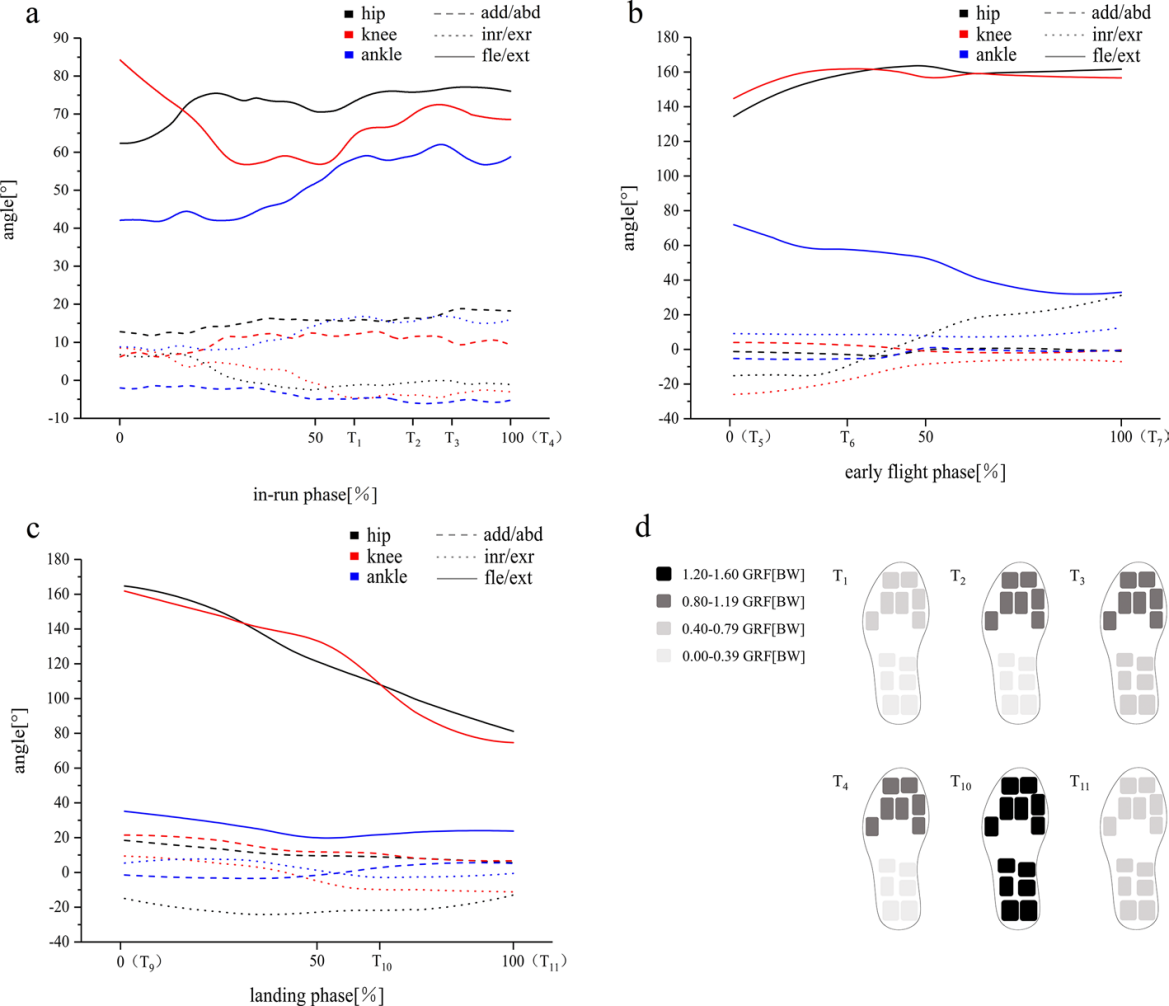
Example data of angle parameter (half of the sum of both sides of the lower limbs, average of eight subjects) from the inrun (**a**), early flight (**b**) and landing phases (**c**) [%, T_1_: straight inrun moment, T_2_: straight turn into arc moment, T_3_: middle of the curve of the inrun moment, T_4_: start of the takeoff moment (end of the inrun moment), T_5_: centre of gravity projection leaving the platform, T_6_: start of early flight moment, T_7_: start of stable flight moment, T_8_: middle of the stable flight moment, T_9_: 0.36 s before the landing moment, T_10_: landing moment and T_11_: landing buffer moment], as calculated using the Xsens system. The data show the changes in the angles of the lower limb joints in different directions of motion. Lineation = adduction/abduction, dotted line = internal/external rotation, solid line = flexion/extension (dorsiflexion/ plantarflexion), black = hip, red = knee, blue = ankle. Graph **d** depicts the plantar pressure distribution at different characteristic times, and colour depth reflects the amount of pressure (the sum of the fore and the rear). Ground reaction force (GRF) is standardised by body weight (half of the sum of both sides of the feet, average of eight subjects)

**Fig. 5:**
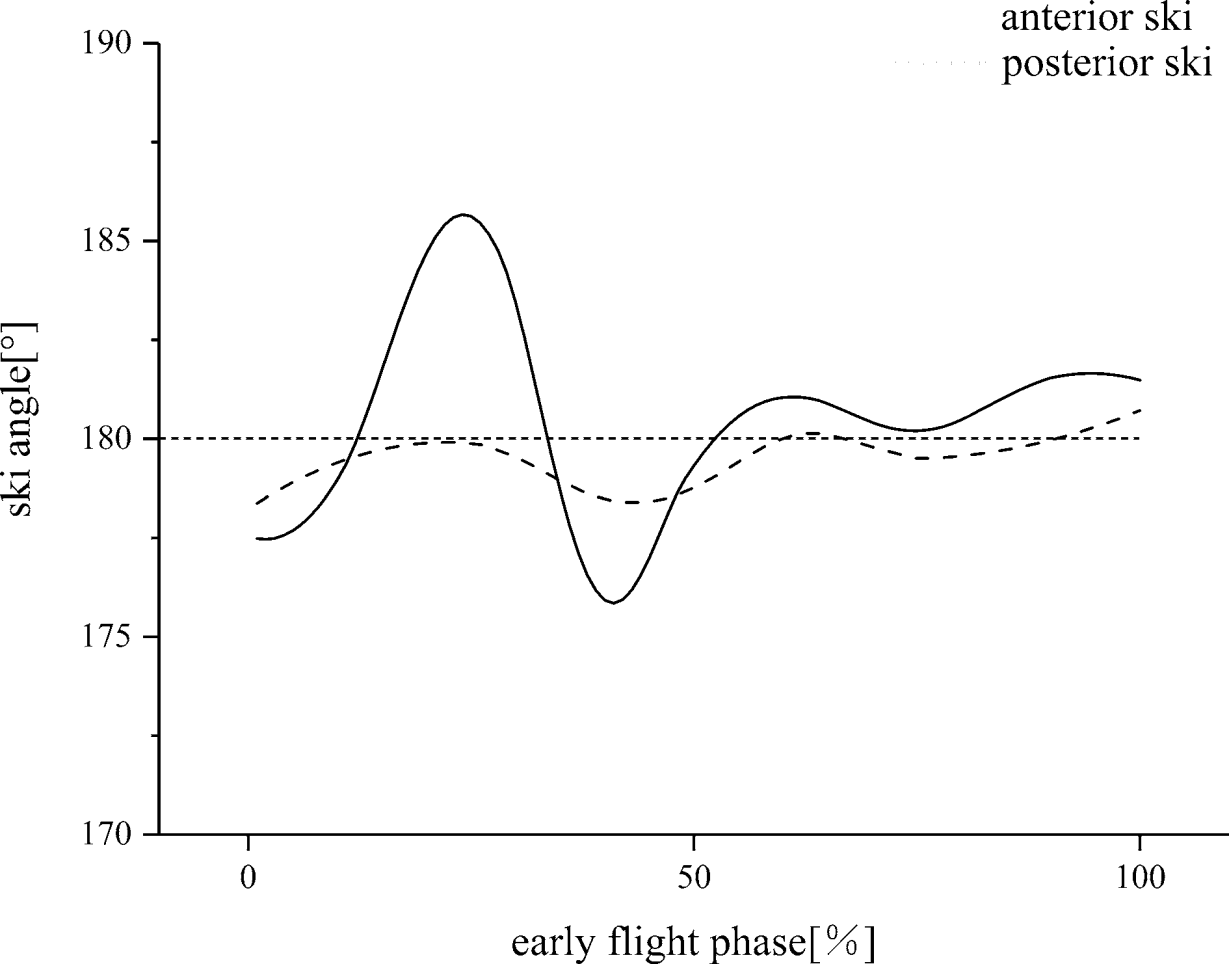
Example data of ski angle parameters (average of eight subjects) from the early flight phase as shot using 2D video (500 Hz) on the left. Points ski-1, ski-2 and foot form the ski anterior angle. Points foot, ski-3 and ski-4 form the ski posterior angle

Figure 3 illustrates the placement of application sensors and the athlete’s body posture during early flight. Figure 4 depicts the changes in kinematics and dynamics of the key transition technology, involving straight turn into arc during the inrun phase, early flight during the flight phase and landing preparation during landing. Figure 5 and Tables 1 and 2 provide the relevant parameters of flight posture and ski movement.

**Table 1:** Key angle parameters of athlete’s body posture during early flight, T_7−0.30 s_ = 0.30 s after the start of the stable flight moment (limited capture range of the camera), data of angle parameters (average of eight subjects) from the early flight phase as shot continuously on the left using the Simi system

Time(s)	α(°)	β(°)	γ(°)
T_6_	62.76 ± 1.89	75.31 ± 5.87	52.84 ± 4.99
T_7_	56.84 ± 0.69	42.17 ± 1.62	29.57 ± 2.16
T_7-0.30 s_	52.31 ± 6.72	28.04 ± 6.66	19.56 ± 6.04

**Table 2:** Key angle parameters of the ski during early flight and landing

Time(s)	Roll(°)	Yaw(°)	Pitch(°)
ΔT_7_	Δ14.85 ± 10.73	Δ12.81 ± 6.63	Δ34.38 ± 16.90
ΔT_8_	Δ1.99 ± 1.02	Δ3.68 ± 1.92	Δ2.54 ± 2.35
ΔT_10_	Δ24.49 ± 8.05	Δ24.23 ± 5.47	Δ37.47 ± 6.47
ΔT_11_	Δ2.35 ± 5.86	Δ3.05 ± 4.62	Δ2.82 ± 3.53

ΔT_7_ denotes the change value between T_6_ and T_7_. The definitions of ΔT_8_, ΔT_10_ and ΔT_11_ are the same as above. Data of angle parameters (average of eight subjects) from the early flight and landing phases as calculated using the Xsens system (measured by the IMU on the ski)

## Discussion

The objective of this study is to investigate the application validity of the Xsens system in ski jumping. This research selects the lower limb joint angle with evident kinematic changes during the takeoff phase, which exerts the greatest effect on performance [18], for analysis. Zhang [19] pointed out that compared with traditional motion capture systems, the Xsens system can accurately obtain the flexion and extension angles of all joints under the condition of daily activities. The validation results of the current study show the excellent application effect of the Xsens system to ski jumping (Fig. 2). Robert [20] asserted that the RMSE of task data processing between systems should be less than 5°, but the calculation result of the present study is slightly larger than the reference standard (Fig. 2). The reasons for the aforementioned differences may be as follows. Firstly, the accuracy of IMUs will be affected by the difficulty and duration of the task. Secondly, the models and frameworks of the two systems are different. The Simi system uses the Hanavan mathematical model (15 simple rigid bodies), whilst the Xsens system uses the MVN model based on the International Society of Biomechanics model. Thirdly, the joint coordinate systems and positions of the two systems are different. Each IMU in Xsens has its own specific position, which is a 3D coordinate system. The anatomical measurement marks in the Simi system are generally the centre of each link or joint, which is a 2D coordinate system in the current research. In summary, the method of applying the Xsens system to ski jumping is proven as feasible, further validating the effectiveness of the Xsens system compared with the data measured by the Simi system. In recent years, many studies have focused on applying IMUs to ski jumping projects [21, 22], proving that this technology exhibits high development potential and application prospects.

The high sampling frequency video (200 Hz, 500 Hz) used in the current study cannot only analyse the data at the end of the experiment, but can also provide quick feedback to coaches and athletes through on-the-spot slow playback. The whole motion animation process of the human body model generated by Xsens can more intuitively deepen the understanding of technical movements of coaches and athletes. The dynamic parameters of the left and right feet collected by the wireless insole can also help athletes in better judging the rationality of the execution accuracy (timing) of lower limb technical actions and whether laterality exists, providing guidance for daily training. The transmission and storage of the aforementioned data are mostly achieved in offline or wireless Bluetooth mode, which will not affect athletes’ movements on the premise of obtaining high-quality data. In future research and development, this combination of IMU, high-speed video camera and wireless insole is suggested as a more convenient and economical scheme for recording the daily training, and even the competitions, of athletes.

On the basis of the biomechanical measurement system established in the current study, the transition process of key technologies in ski jumping is analysed as follows. *Straight turn into arc in the inrun phase*: in the straight area, athletes can achieve a streamlined posture of ‘rolling up’ by stretching the shoulder, elbow and wrist joints to the rear of the body, and then pressing them close to both sides of the body, followed by flexing the hip, knee and ankle joints, and finally, leaning forward to lower the centre of gravity. This posture meets the requirements of fluid mechanics and can minimise air resistance. After forming a stable posture, an athlete’s lower limb joint angle is always maintained within a certain range, even at the transition point (Fig. 4a). Janura [23] recorded the sliding posture of 656 male ski jumpers throughout a 10-year longitudinal study and found that the angle of an athlete’s lower limb joints tended to be generally stable, with only a slight change, which may be related to the change in sliding technology or clothing. Combined with the results of the current study, no evident change in kinematics is shown after athletes have formed a stable posture.

More changes occur in dynamics, particularly at the transition point of sliding (Fig. 4d T_2_, T_3_). In the straight area, the size of the GRF of athletes remains nearly unchanged, and the body is under a static condition of low load, which is the result of the dynamic balance between the opposing and positive torques. Centripetal force will be generated after entering the entrance of the arc area (the first transition point). GRF increases from 0.76 BW of T_1_ to 1.12 BW of T_2_, producing a large opposing torque, which forces athletes to strengthen the control of each joint muscle and increase positive torque. At this moment the body will enter a high load control status. GRF reaches the maximum value of 1.47 BW at T_3_, which is primarily related to speed, platform gradient and radius. Ettema [24] designed a rigid dummy model to simulate the state of athletes’ sliding and then determined that GRF would increase from 0.88 BW to 1.65 BW after entering the arc area. The current study further verified Ettema’s calculation results through field tests. At T_4_, GRF dropped to 1.09 BW. At this moment, the athletes are about to enter the takeoff area close to the level, and then, additional centripetal force will disappear. To summarise, each joint muscle group of an athlete should have a highly coordinated and fast variable load control ability to adapt to this dynamic change, because this dynamic change is related to acceleration, which changes the final velocity of sliding (the initial velocity of takeoff), and thus affect jumping distance.

At present, the sudden increase in centripetal force at the entrance of the arc area is generally believed to be not conducive to the maintenance of motion during the sliding process. The International Ski Federation and scholars have given attention to this phenomenon and proposed some solutions, such as applying the third power function, cosine function and iterative shooting method to the geometric structure of the arc area of the slideway [25, 26]. This change can effectively avoid the instantaneous increase in curvature and slowly increase the reaction force endured by athletes under the condition of ensuring the same sliding speed, which optimises the original sliding system.

The limitations of the wireless pressure insole in this study are worth mentioning. Firstly, the collected data only represent the surface (vertical direction) relative to the insole. In accordance with statistics, only about 80% of the maximum GRF can be collected [9], which is inevitable. Secondly, in similar studies, some scholars stated that sampling frequency may exert a certain effect on the results, but others think that sampling frequency will not or will only slightly affect the results [27, 28]. The current study found that the applied pressure insole can obtain the plantar pressure parameters at the characteristic time. However, given the low sampling frequency (50 Hz), some data are discontinuous during takeoff and landing, which may be caused by the high acceleration generated in the two phases. A pressure insole with a high sampling frequency (about 200 Hz) is suggested to be used in subsequent studies.

*Early flight in the flight phase*: as shown in Fig. 4b, after the projection of an athlete’s centre of gravity leaves the takeoff table (T_5_ and T_6_), the athlete still maintains the activities of extending the hip and knee in the takeoff phase and further fully stretches the hip joint (about 160°), knee joint (about 160°) and dorsiflex ankle joint (about 40°) during the early flight phase (T_6_ and T_7_). In this process, the refined manipulation of the athletes’ lower limbs is mostly external rotation and abduction, and the degree of hip and knee external rotation is relatively large. Table 1 provides the key angle changes of an athlete’s body posture during early flight (T_6_ and T_7_) and after stable flight (T_7−0.30 s_). In the process, an athlete leans forward as boldly as possible (α, 63° to 52°) and close to the ski (β, 75° to 28°; γ, 53° to 20°) (Fig. 3b). Some scholars [29] stated that the best conditions for early flight are a small body angle of attack, a body lean angle, a ski angle of attack and time to reach a stable flight. Gardan [30] and Marqués-Bruna [31] pointed out from the perspective of aerodynamics that the larger the body lean angle, the greater the pressure difference between the upper and lower parts of the athlete, and the athlete will obtain greater lift. Simultaneously, a smaller body lean angle can effectively reduce inertial vibrations during flight, increasing flight stability in the air and thus achieving larger flight distance. Therefore, quickly and accurately completing the corresponding technical actions (particularly more boldly leaning forward) during the early flight phase is particularly important.

Adjusting the ski’s angle for the first time after the athlete’s takeoff is crucial [13]. The current study firstly quantified the deformation characteristics of the skis after the takeoff through video capture with high sampling frequency (500 Hz). As shown in Fig. 5, ski deformation occurred when the projection of the centre of gravity of an athlete leaves the takeoff table and is basically at the position of the ski holder (57% of the ski length). The fore end of the ski firstly produces downward longitudinal deformation, then upward and finally disappears gradually. The maximum change of the angle is about ± 5°. The rear end of the ski is deformed after the ski structure completely leaves the takeoff table. The change is basically similar to that of the fore end of the ski (nearly the same frequency), but the longitudinal deformation is small, and the maximum angle change is about ± 2°. The reasons for such deformation characteristics may be as follows. The first reason is the mechanical changes of the ski. When the projection of the body’s gravity centre leaves the table, the support force (FN) of the table will be gradually lost from the fore end to the rear end of the ski. The fore end will bear the downward gravity G of the body, leading to the downward trend of the fore end of the ski before the rear end, until the ski structure completely leaves the table. The rear end of the ski will also lose the remaining FN, resulting in a downward trend. The second reason is the material structure of the ski. A special ski is different from the general freestyle snowboard. Its length is about 147% of an athlete’s height, the board surface is longer and wider and the fore end of the ski is significantly longer than its rear end. To achieve better gliding effect, controlling the weight of the ski, which should not be excessively heavy, is necessary. The lightweight and high-strength multistage structure in the compatibility of ski materials is bound to reduce the overall hardness (stiffness) of the ski and increase its elastic properties. Some aerodynamic characteristics of ski deformation should be given attention in future research, and the relationship between this deformation and sports performance should be explored further.

Marqués-Bruna [32] stated that the athlete-ski system is also applicable to the principles of aeronautics in air flight, primarily involving the cross-coupling of pitch, roll and yaw. In the current study, the pitch, roll and yaw of a ski in the early flight phase are further obtained from the IMU placed on the ski (Fig. 3a). The data in Table 2 indicate that the pitch angle change of the ski is about 34°, roll is 15° and yaw is 13° during the early flight phase (ΔT_7_). In this process, athletes mainly rely on the external rotation and abduction of the lower limb joints, and the ankle plays a leading role in controlling the ski to form a ‘V’ shape with flight advantages, achieving better aerodynamic effect. However, after reaching a stable attitude (ΔT_8_), the pitch, roll and yaw angle changes of the ski tend to be stable, i.e. about 2°, 4° and 3°, respectively, and can only be adjusted slightly due to changes in air resistance or wind direction to maintain the relative stability of flight posture. The test results prove the application effect of IMU to ski jumping research, particularly in monitoring the composite movement of the ski. This method can be used as a routine training monitoring method to help coaches judge the quality of the flight skills of athletes.

*Landing preparation in landing phase*: researchers and athletes give minimal attention to the landing phase, and only a few biomechanical studies have been dedicated to the landing phase [33]. Research on the landing phase is limited to the initial stage of the study. However, the development and application of wireless connectivity and sensors in the past few years have provided a new means of researching the landing phase. Veronica team [9, 11] explored the combination of wireless pressure insole and IMU to study different landing technologies and their effects on landing impact, proving the effectiveness of this method.

On the basis of the aforementioned methods, the current study captured the landing performance of athletes during the landing phase. In this process, the athletes mostly change the ‘V’-shaped posture of flight into the postures of landing preparation and landing buffer. As shown in Fig. 4c, the athletes perform hip flexion and knee flexion in advance before landing (T_9_ and T_10_), further increasing hip flexion (about 80°) and knee flexion (about 70°) after landing (T_10_ and T_11_) to cushion landing impact. In the landing preparation phase, the athlete’s refined manipulation of the lower limbs is contrary to that during air flight, mostly to recover the external rotation and abduction limbs and actively prepare for a smooth and positive landing. In the process of landing buffering to maximum buffering, the athletes in the current study adopted the technique of parallel landing with their feet (squatting). The athletes averaged the impact force of landing on both sides of their lower limbs and absorbed it with full feet. The maximum GRF of T_10_ can reach 2.7 BW, and the GRF of T_11_ can be absorbed to 1.4 BW, which is within the normal sustain range of the human body (Fig. 4d, T_10_ and T_11_). The reasons for the smaller impact may be as follows. Firstly, the size of the takeoff table is small and its height is low. The second reason is lift effect in flight. Lift always holds the athletes to glide, making the landing situation different from other snow sports with great impact on landing, such as freestyle aerials and big air.

In fact, ski jumpers have to use Telemark to land, which is similar to a bow stance or step. Thus, they can obtain technical scores from the referee in the landing phase. From the perspective of safety, however, Telemark technology will cause one knee joint to bear higher GRF and increase the risk of lower limb injury, particularly the injury of the anterior cruciate ligament. Therefore, athletes must make a trade-off between technical score and safety.

The aerodynamic changes experienced by objects flying close to the ground are called ground effect, and the influence of this phenomenon on sport performance is rarely given attention to in empirical research. Seo [34] found in a wind tunnel experiment that a good ski position in the landing preparation phase can increase jumping distance by up to 3 m. A larger angle between the ski and airflow cannot only improve the air lift effect, but also achieve better cushioning effect in the landing phase. However, this monitoring method is uneconomical in training, and the method of placing IMUs on skis seems more accurate in monitoring changes in skis. This method has been applied in other snow research, such as cross-country skiing [35] and ski mountaineering [36]. Table 2 presents the angle changes of the skis on the three axes during and after landing preparation. In the landing preparation process, athletes largely rely on the internal rotation and adduction of the lower limb joints, and the ankle plays a leading role in controlling the skis to recover from the ‘V’ shape in stable flight to the parallel state (ΔT_10_). The ski changes more in pitch about 37°. The angle of the ski exhibits nearly no change (ΔT_11_) after landing and taxiing on the land slope. In summary, combined with the research results of Bessone [14], pitch is the key factor that affects GRF and landing sport performance. Athletes should give attention to the preparation before landing, particularly the pitching of skis. Before landing, ground effect should be used as much as possible to extend flight distance and improve the stability and safety of landing.

## Conclusion

The combination of 2D high-speed video recording, IMU and wireless pressure insole can capture the sports performance of ski jumpers within a large range in the outdoor field without affecting the athletes’ execution of technical actions. The research results prove that the measurement system is effective and exhibits certain application prospect.

The established measurement system can effectively capture the key transition technical characteristics of ski jumpers whilst observing the overall movement stage, particularly the dynamic changes of the straight turn into arc in inrun, the adjustment of body posture and ski movement in early flight and landing preparation.

Researchers should give more attention to the key transition technologies of ski jumping, and further research should focus on the effects of these key transition technologies on the sports performance of ski jumpers. Athletes and coaches should place importance on key transition technologies and conduct special training in accordance with different technical needs to improve the sports performance of athletes.

## Data Availability

The datasets used and/or analyzed during the study are available from the corresponding author on reasonable request.

## Abbreviations

IMUs: Inertial measurement units
RH: Relative humidity
RMSE: Root-mean-square error
GRF: Ground reaction force
FN: Support force
